# Cocaine-induced Type-A Aortic Dissection Extending to the Common Iliac Arteries

**DOI:** 10.7759/cureus.2059

**Published:** 2018-01-12

**Authors:** Mohamed A Mohamed, Rohit Abraham, Tareq I Maraqa, Samir Elian

**Affiliations:** 1 Michigan State University College of Human Medicine; 2 Trauma Department, Hurley Medical Center, Michigan State University College of Human Medicine; 3 Cardiology, Hurley Medical Center

**Keywords:** cocaine complications, aortic dissection, aortic emergencies, cardiovascular emergencies

## Abstract

Aortic dissection is a rare and fatal complication of cocaine-induced hypertension. The injury mechanism is through shear stress that penetrates the intimal vessel layer, allowing blood flow to separate intimal and medial layers. Due to its scarcity and the paucity of related literature, our knowledge of this condition is limited. We present a rare case of a cocaine-induced aortic dissection, which extended continuously from the aortic root to the common iliacs, along with a literature review of similar cases.

A 48-year-old male with recent cocaine use presented with left-sided chest-pain, which radiated to the back with nausea, diaphoresis, and shortness of breath. The patient was hypotensive. The initial radiographs and computed tomography were negative. The cardiac enzymes were elevated and the patient was admitted to rule out acute coronary syndrome. Next day echocardiogram and computed tomography revealed a Type-A aortic dissection continuously extending from the aortic root to the left common iliac artery. The patient was immediately transferred for surgery. Postoperatively, he developed acute kidney injury and shock liver. The patient status continued to deteriorate and he expired on postoperative day four.

This case demonstrates the importance of prompt and thorough diagnostic evaluation, despite subjective history and initially negative imaging that might point towards other conditions. Unlike the previous cases, our case failed to identify the positive history of cocaine until nearly 24 hours into the patient’s hospital course, suggesting a need for close monitoring in these patients and a possible need for repeat imaging.​​​​​​​

## Introduction

Cocaine continues to be the most common illicit substance used that prompts emergency department (ED) visits in the United States (US) [1]. Cocaine-induced complications are several, but cocaine-induced aortic dissection (CIAD) is a grave cardiovascular complication associated with a 40% mortality rate [2]. The exact incidence of CIAD is uncertain. However, up to 0.5% of ED visits for chest pain with a history of cocaine use have been reported to develop CIAD [3]. With the current epidemic observed in the US, clincians should be cognizant of rarer cocaine-related complications that may be increasingly observed. Herein, we present a rare case of a 48-year-old man who developed a Stanford Type-A CIAD, which extended continuously from the aortic root to the common iliacs.

## Case presentation

A 48-year-old male presented to the ED complaining of a 7/10 left-sided chest pain that began 40 minutes prior. The pain radiated to the back, jaw, and left arm and was associated with nausea, diaphoresis, and dyspnea. The pain was alleviated only when supine, but relief was transient. His pertinent past medical history included recent cocaine use, hypertension, a 30-pack-year smoking history, hypercholesterolemia, and Type-II Diabetes Mellitus.

**Figure 1:**
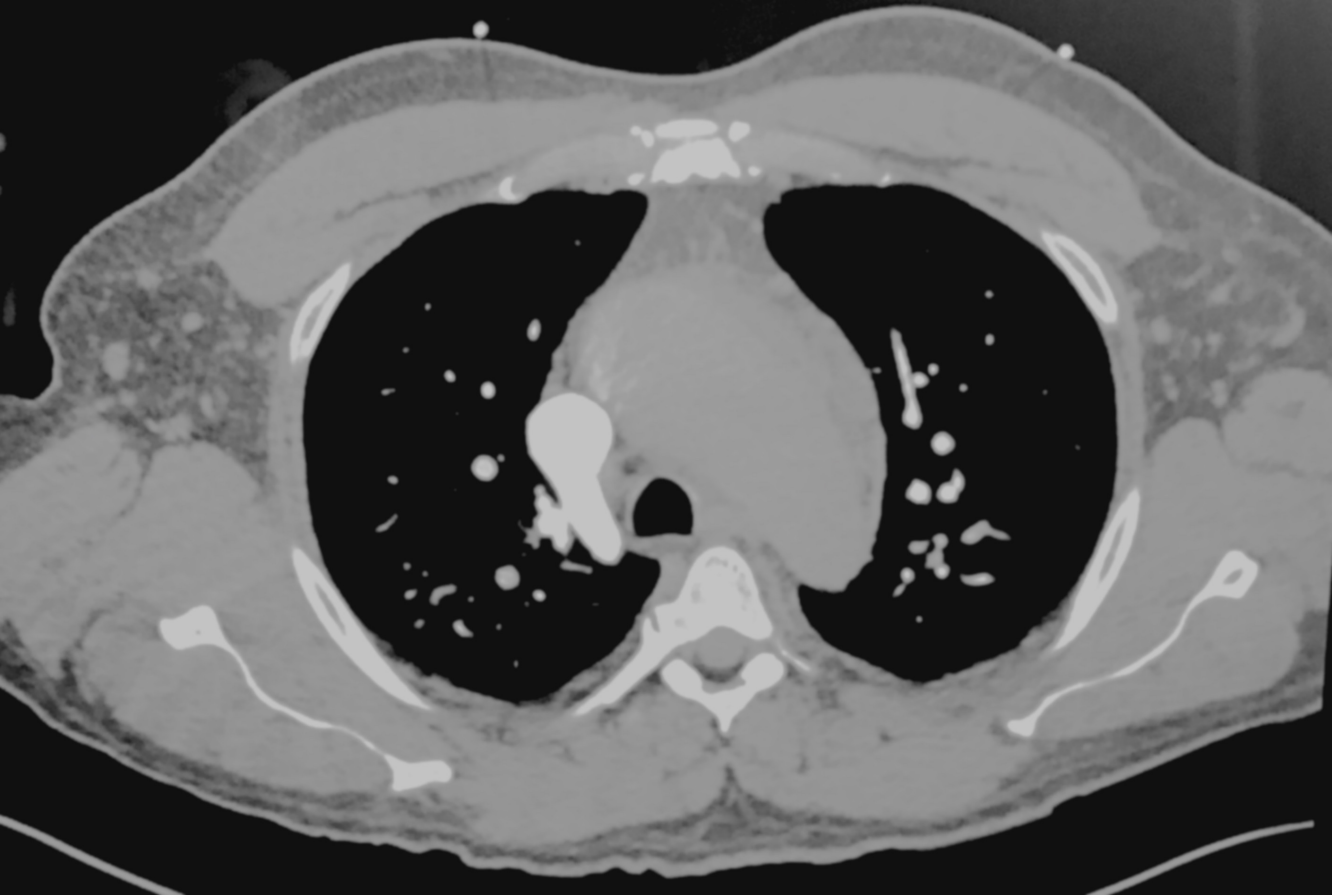
Initial transverse computed tomography (CT) lacking signs suggestive of acute aortic dissection

His vitals were as follows: blood pressure (BP) from the right arm 101/34 mmHg; heart rate 81 beats/min; respiratory rate 20 breaths/min; and SaO2 94% on room air. His physical examination was mostly unremarkable. An initial chest radiograph and computed tomography (CT) with contrast were negative for dissection (Figure 1). An electrocardiogram (ECG) revealed ST-depressions and deep T-wave inversions in leads V4-V6, suggestive of a non-ST-elevation myocardial infarction (NSTEMI). The patient was started on intravenous (IV) fluids, low-dose nitroglycerin, aspirin, and continuous heparin infusion. The patient was admitted per acute coronary syndrome protocol and for further monitoring.

**Figure 2:**
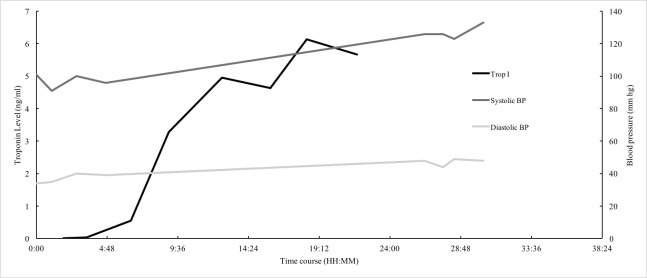
Time course of Troponin-I and blood pressure

**Figure 3:**
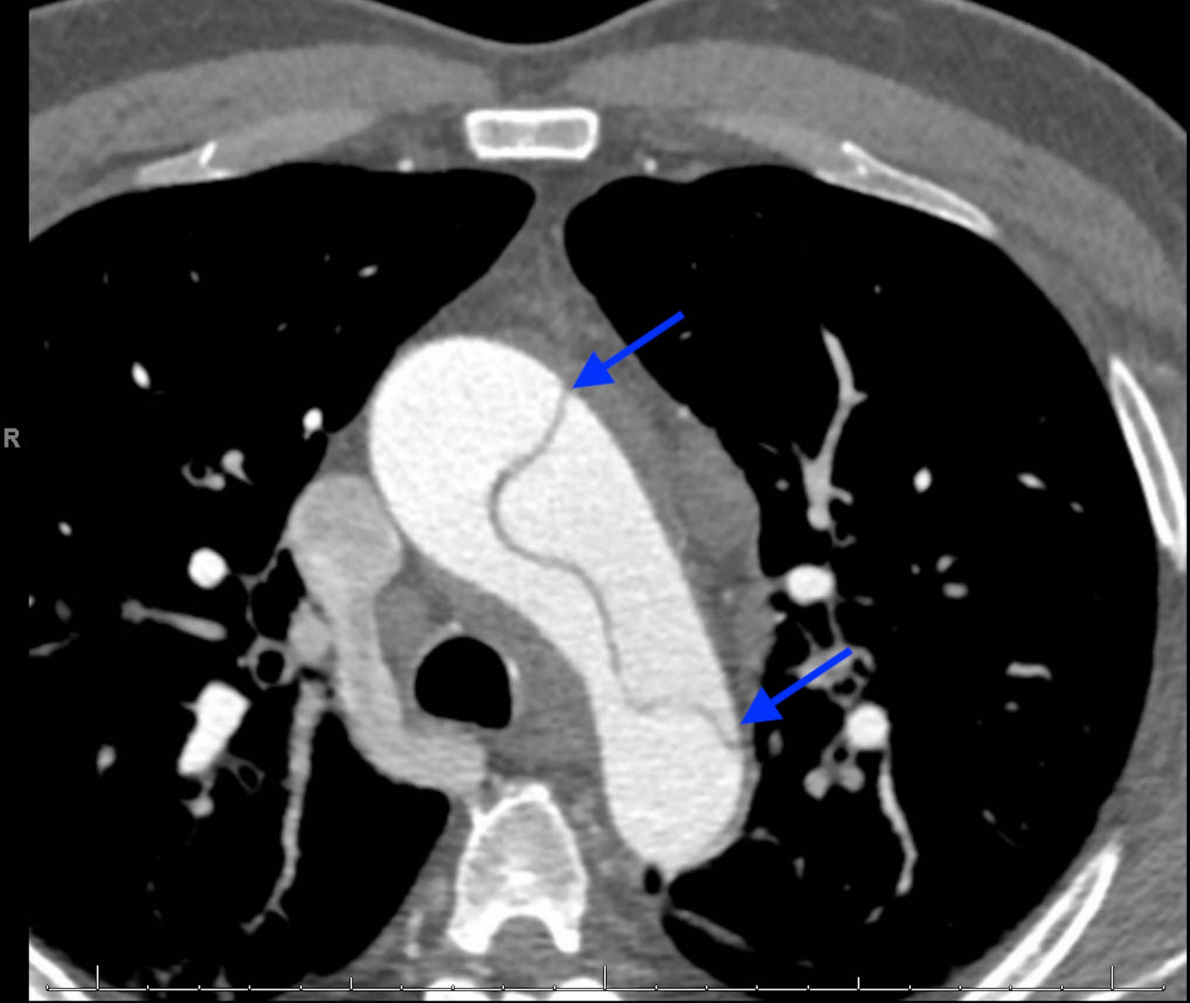
Transverse section demonstrating the dissection along the entire span of the aortic arch

**Figure 4:**
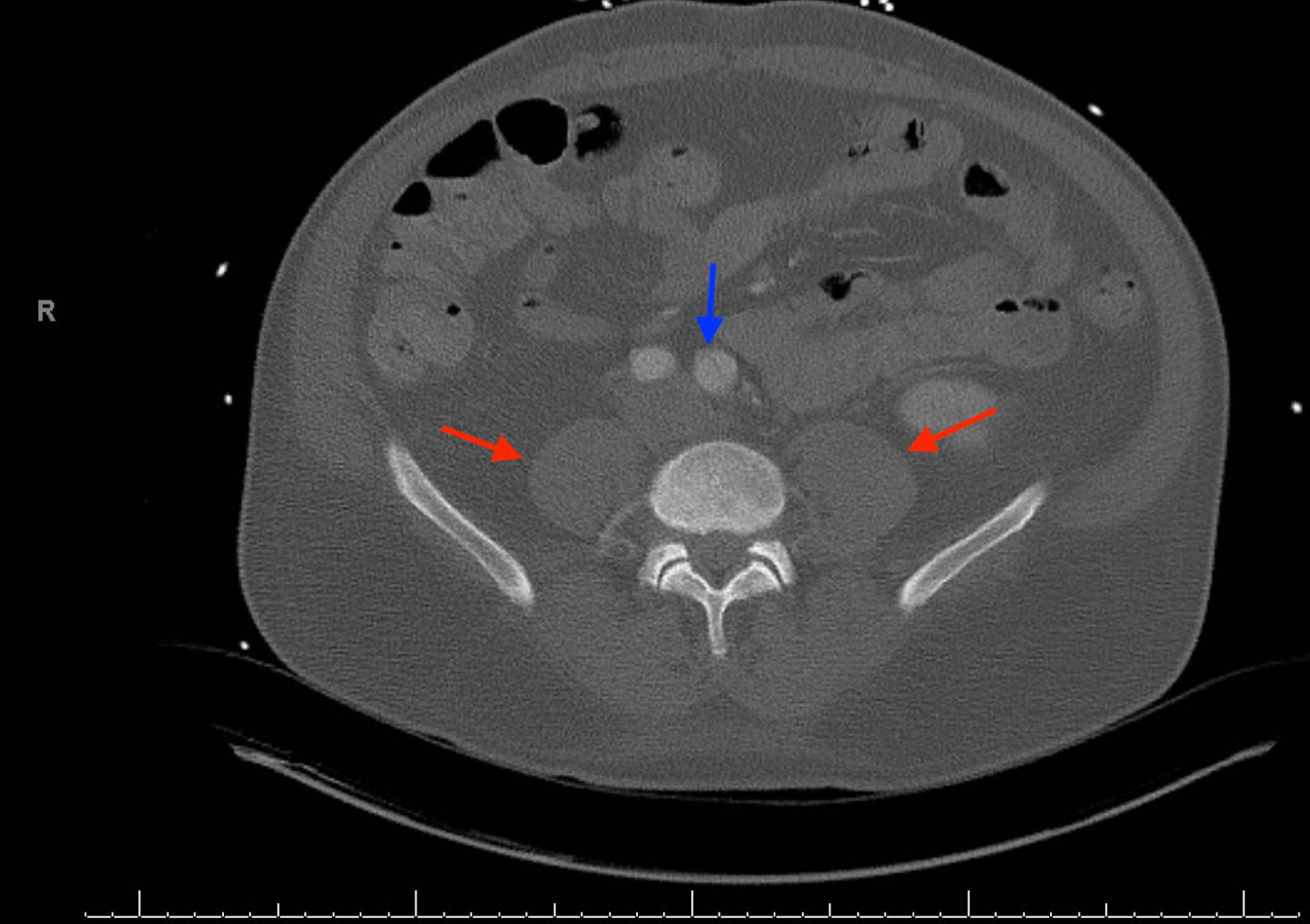
Transverse section demonstrating the distal extension of the dissection (blue arrow) at the level of the psoas muscles (red arrows)

**Figure 5:**
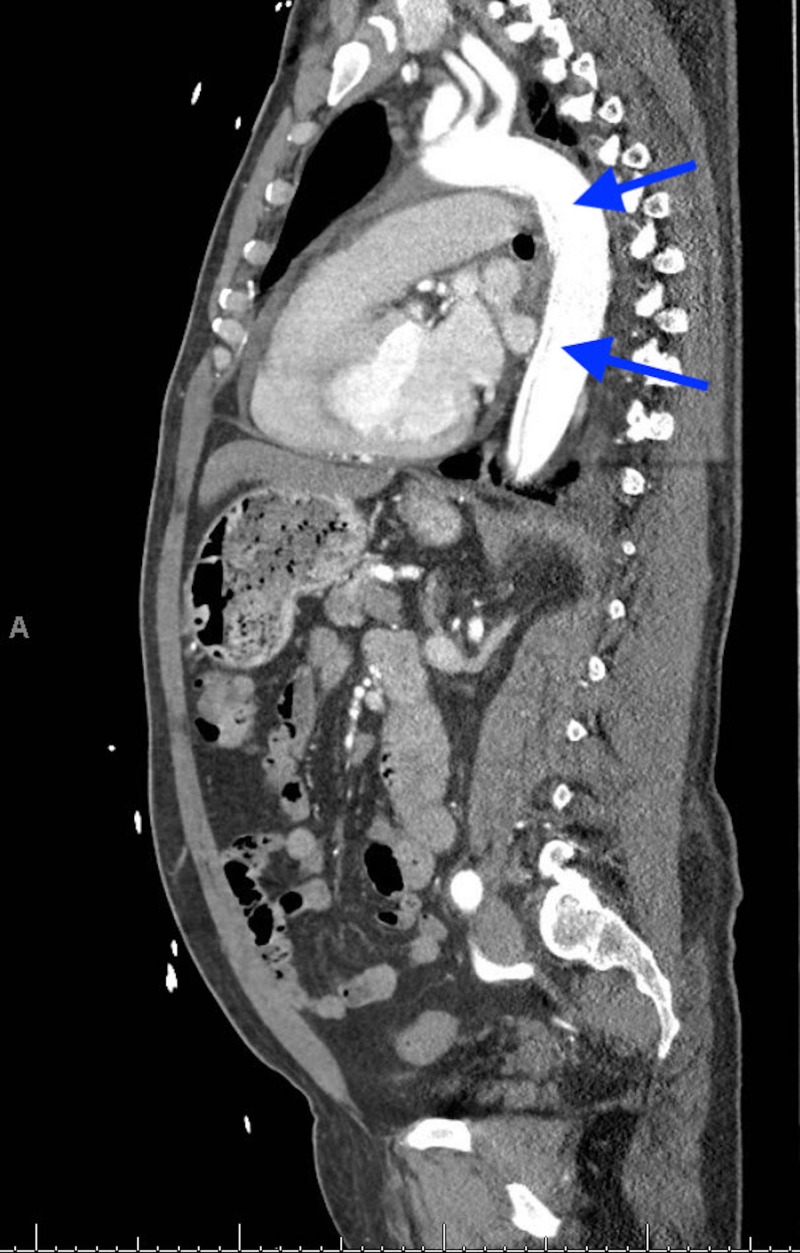
Frontal section demonstrating the acute dissection along the thoracic aorta (blue arrows)

Troponin levels began increasing the following day, despite resolution of the chest pain and BP stabilization (Figure 2). A urine drug screen was positive for cocaine. Due to leukocytosis and history of illicit drug use, a transesophageal echocardiography (TEE) was performed to rule out valvular dysfunction. The findings were suggestive of severe aortic root dilatation and aortic regurgitation without vegetation but was suspicious for acute aortic dissection (AAD). Subsequent computed tomography angiography (CTA) revealed a Stanford Type-A dissection, originating proximally from the aortic root extending distally to the left common iliac artery(Figures 3-5).

Anticoagulant therapy was immediately discontinued, and the patient was started on esmolol and fenoldopam for BP control. He was transferred for emergent surgical intervention and underwent aortic fenestration with a left renal stent. On postoperative day 3 (POD-3), repeat imaging revealed that the CIAD intimal flap had extended cranially to the maxillary artery. Subsequently, he developed cardiac tamponade prompting mediastinal exploration, which was further complicated by acute kidney injury and shock liver. The patient status continued to deteriorate and he expired on POD-4.

## Discussion

This case represents a rare and peculiar presentation of CIAD, extending beyond the complete range of the aorta. Acute aortic dissection is not an infrequent condition, with an incidence of up to 3.5 per 100,000 person years. However, AAD outcomes are grave and associated with high morbidity and mortality. The classical clinical manifestation of AAD is sudden, severe chest pain radiating to the back or abdomen, often described as ripping or tearing [2]. AAD most commonly affects elderly males with a median age of 62 [4]. However, younger patients are often the patients that present with CIAD, likely attributed to ages by which cocaine is most commonly used. Several risk factors predispose individuals to AAD (Table 1)[2]. An AAD in younger patients should prompt further evaluation for associated congenital cardiovascular anomalies [5]. Our patient’s young age precluded a chronic nature to his hypertension and smoking, his aortic media were additionally weakened by polytrauma and surgical history, and his initial ECG showed ST-elevations in the inferior leads.

**Table 1:** Summary of risk factors of acute aortic dissection

Physical risk factors
Marfan syndrome
Bicuspid aortic valve
Aortic aneurysm
Previous heart surgery
Trauma
Hypertension
Behavioral risk factors
Smoking
Cocaine use

As a potent vasoconstrictor, cocaine increases the risk for AAD. Cocaine-induced shear stress penetrates the intimal vessel layer and allows blood to separate both the intimal and medial layers. This may take hours to days to occur and initial imaging may be negative, similar to our patient [6]. Our patient’s initial presentation was negative and about 38% of AADs are initially missed on imaging [7]. Moreover, 21% of AAD cases receive anticoagulation therapy, which can cause further complications, with associated mortality rates increased up to 44% [8]. In our patient, high doses of anticoagulants were avoided, despite misleading initially negative imaging, by clinical signs that prompted CT angiography that diagnosed his Type-B AD, which was medically managed [9]. Therefore, a high index of suspicion for CIAD should be prompted, particularly when younger males present with early clinical signs and symptoms of severe or tearing chest pain, acute hypo/hypertension, pulse deficits, or widened mediastinum.

The Stanford classification system is the most commonly used system, and immediate classification is necessary to determine management of AAD [4]. Stanford Type-B ADs are medically managed by stabilization of BP and rate, while Type-A requires similar stabilization initially preceded with emergent surgical repair of the ascending aorta. An international cohort study showed that both Types A and B can be induced by cocaine and the distribution is similarly seen in non-cocaine users [3]. Our patient recovered though swift surgical repair, despite anticoagulative therapy provided prior to clinical manifestation of the patient’s evolving AD.

In the past two decades, Stanford Type B mortality has decreased [4]. This improvement in mortality rate could be attributed to better recognition and more efficient diagnosis, which is typically made by CT or bedside transesophageal echocardiography (TEE). Furthermore, a urine drug screen should always be performed in these patients to rule cocaine-association. Additionally, a bedside TEE revealed another cocaine-induced Type A AAD, which was also complicated by severe aortic regurgitation.

To our knowledge, from a cursory literature review spanning 30 years, we believe that our patient suffered from one of the most extensive cases of CIAD. Fortunately, our patient shared a positive outcome following surgical repair of the ascending aorta and aortic valve replacement. Correale et al. documented a case of cocaine-induced AD in a 24-year-old male with a similar initial ED presentation of hypotension and acute coronary syndrome with risk factors of cocaine abuse and smoking [10]. While the association of cocaine and longer dissections has not previously been published, it is plausible that cocaine’s mechanism of action leads to longer dissections on average than in non-cocaine users.

## Conclusions

Our case demonstrates the importance of prompt and thorough diagnostic evaluation, despite subjective history and initially negative imaging that might point towards other conditions. Unlike the previous cases, our case failed to identify the positive history of cocaine until nearly 24 hours into the patient’s hospital course. Immediate urine drug screen upon ED admission would have more rapidly led us towards a diagnosis of AD. However, our patient’s paradoxical initial presentation of hypotension fortunately steered us away from beta blocker therapy—classically causing unopposed alpha-induced vasoconstriction in cocaine abuse—which would have created catastrophic complications in our patient’s evolving AD.

## References

[1] Crane EH (2013). Update on Drug-Related Emergency Department Visits Attributed to Intentional Poisoning: 2011. The CBHSQ Report. Center for Behavioral Health Statistics and Quality, Substance Abuse and Mental Health Services Administration.

[2] Mehta RH, O'Gara PT, Bossone E (2002). Acute type A aortic dissection in the elderly: clinical characteristics, management, and outcomes in the current era. J Am Coll Cardiol.

[3] Dean JH, Woznicki EM, O'gara P (2014). Cocaine-related aortic dissection: lessons from the International Registry of Acute Aortic Dissection. Am J Med.

[4] Barth CW, Bray M, Roberts WC (1986). Rupture of the ascending aorta during cocaine intoxication. Am J Cardiol.

[5] Ngan KW, Hsueh C, Hsieh HC, Ueng SH (2006). Aortic dissection in a young patient without any predisposing factors. Chang Gung Med J.

[6] Schwartz BG, Rezkalla S, Kloner RA (2010). Cardiovascular effects of cocaine. Circulation.

[7] Khan IA, Nair CK (2002). Clinical, diagnostic, and management perspectives of aortic dissection. Chest.

[8] Shah R, Berzingi C, Fan TH, Askari R, Khan MR (2015). Cocaine-induced acute aortic dissection. J Emerg Med.

[9] Goldberg A (2015). Superimposed cocaine-induced rhabdomyolysis in a patient with aortic dissection rhabdomyolysis. A A Case Rep.

[10] Correale M, Di martino L, Ieva R, Di biase M, Brunetti ND (2011). Aortic dissection after cocaine abuse. Clin Res Cardiol.

